# Encephalitis and Death in Wild Mammals at a Rehabilitation Center after Infection with Highly Pathogenic Avian Influenza A(H5N8) Virus, United Kingdom

**DOI:** 10.3201/eid2711.211225

**Published:** 2021-11

**Authors:** Tobias Floyd, Ashley C. Banyard, Fabian Z.X. Lean, Alexander M.P. Byrne, Edward Fullick, Elliot Whittard, Benjamin C. Mollett, Steve Bexton, Vanessa Swinson, Michele Macrelli, Nicola S. Lewis, Scott M. Reid, Alejandro Núñez, J. Paul Duff, Rowena Hansen, Ian H. Brown

**Affiliations:** Animal and Plant Health Agency, Weybridge, UK (T. Floyd, A.C. Banyard, F.Z.X Lean, A.M.P. Byrne, E. Whittard, B.C. Mollett, S.M. Reid, A. Núñez, R. Hansen, I.H. Brown);; Animal and Plant Health Agency, Thirsk, UK (E. Fullick, V. Swinson);; Royal Society for Prevention of Cruelty to Animals, East Winch, UK (S. Bexton);; Animal and Plant Health Agency, Bury St Edmunds, UK (M. Macrelli);; Royal Veterinary College Department of Pathobiology and Population Sciences, North Mymms, UK (N.S. Lewis);; Animal and Plant Health Agency Diseases of Wildlife Scheme, Penrith, UK (J.P. Duff)

**Keywords:** avian influenza, systemic infection, first detection, terrestrial carnivores, cetaceans, H5N8, highly pathogenic, United Kingdom, viruses, respiratory infections, zoonoses

## Abstract

We report a disease and mortality event involving swans, seals, and a fox at a wildlife rehabilitation center in the United Kingdom during late 2020. Five swans had onset of highly pathogenic avian influenza virus infection while in captivity. Subsequently, 5 seals and a fox died (or were euthanized) after onset of clinical disease. Avian-origin influenza A virus subtype H5N8 was retrospectively determined as the cause of disease. Infection in the seals manifested as seizures, and immunohistochemical and molecular testing on postmortem samples detected a neurologic distribution of viral products. The fox died overnight after sudden onset of inappetence, and postmortem tissues revealed neurologic and respiratory distribution of viral products. Live virus was isolated from the swans, seals, and the fox, and a single genetic change was detected as a potential adaptive mutation in the mammalian-derived viral sequences. No human influenza-like illness was reported in the weeks after the event.

An episode of unusual disease resulting in deaths in different species at a wildlife rehabilitation center in the United Kingdom during late 2020 led to the retrospective detection of influenza A virus subtype H5N8 of avian origin in 5 mute swans, a fox, and 3 seals. The wildlife rehabilitation center admits >6,000 animals each year. New arrivals are initially housed in a quarantine facility upon admission. Four juvenile common seals (*Phoca vitulina*), 1 juvenile gray seal (*Halichoerus grypus*), and 1 juvenile red fox (*Vulpes vulpes*) died or were euthanized over a 2-day period. The fox died suddenly after a short period of nonspecific malaise and inappetence. The seals exhibited sudden-onset neurologic signs, including seizures before death or euthanasia (Figure 1).

**Figure 1:**
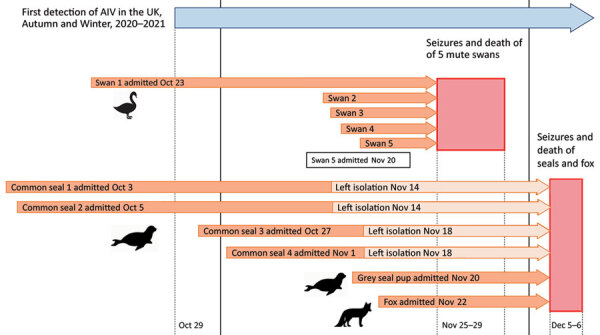
Timeline of the disease event, in which encephalitis and death in wild mammals at a rehabilitation center occurred after systemic infection with highly pathogenic avian influenza A subtype H5N8, United Kingdom. AIV, avian influenza virus; UK, United Kingdom.

This mortality event occurred ≈1 week after the deaths or euthanasia of 5 mute swans (*Cygnus olor*) held in isolation at the center because of acute-onset malaise and terminal seizures. The 5 swans were submitted for examination and testing under the Avian Influenza Wild Bird Surveillance Scheme (undertaken by the United Kingdom’s Animal and Plant Health Agency) (*1*), and they tested positive for highly pathogenic avian influenza A(H5N8) virus.

The unusual spatiotemporal cluster of unexplained death and neurologic disease in multiple avian and nonavian species warranted further investigation. Influenza of avian origin was not suspected in the fox and seals, and none of the other captive birds at the center showed any clinical signs of disease. The linkage between the mortality event in the swans and that observed in the fox and seals was not made until many weeks after the event, when the fox and seal tissues were assessed to attempt to define an etiologic diagnosis.

## Methods

### Postmortem Examination, Tissue Sampling, and Histopathologic Investigation

We collected a thorough clinical history from the rehabilitation center. We subjected the carcasses of 3 of the 5 swans, the fox, and 3 of the 5 seals (1 gray seal and 2 common seals) full postmortem examination (PME). Two swans were submitted frozen and did not undergo PME, but we took oral and cloacal swab samples from these carcasses for virologic testing. We examined plain swab samples from the oropharynx and cloaca of each of the swans, and similarly from the nasal cavity and rectum of each of the 3 seals, for preliminary influenza virologic testing. After receipt of the avian influenza virus results, we sampled the 3 freshest seal carcasses because they were more likely to yield meaningful results and have suitable samples for histopathologic examination. We took tissue samples of brain, liver, kidney, spleen, lymph nodes, and lung from the seals and the fox and stored them at −80°C until required.

For histologic examination, we took samples of heart, lung, liver, spleen, and brain tissues from the swans, fox, and seals, as well as lymph nodes of the mammalian species, and fixed them in 10% neutral buffered formalin before processing for hematoxylin and eosin staining and immunohistochemical (IHC) examination. We performed IHC examination by using anti–influenza A nucleoprotein primary antibody (Statens Serum Institute, https://en.ssi.dk), as previously described (*2*), and anti–canine distemper virus nucleoprotein mouse monoclonal primary antibody (Bio-Rad, https://www.bio-rad.com) in selected fox tissues.

### Virologic Investigation

We assessed swabs taken from the swans and seals and tissue samples taken from the seals and fox for influenza A nucleic acid by using a screening real-time reverse transcription PCR (rRT-PCR) assay (*3*), followed by an H5 subtype–specific rRT-PCR assay (*4*–*6*). Where the subtype-specific rRT-PCR assay detected positive samples, we determined the hemagglutinin cleavage-site sequence (*7*).

We performed virus isolation on PCR-positive samples from the swans, seals, and fox by using specified pathogen-free embryonated fowls’ eggs (*8*,*9*). We used the 3 viral isolates recovered to generate whole-genome sequence (WGS) data by using a MiSeq platform (Illumina, https://www.illumina.com) (*10*). For comparative genetic analysis, we downloaded recent H5 2.3.4.4b virus hemagglutinin sequences from the GISAID EpiFlu database (https://platform.gisaid.org). We deposited sequences in the GISAID database (accession nos. EPI_ISL_1123360, EPI_ISL_2081527, and EPI_ISL_2081528).

Differential diagnosis for the fox samples included molecular testing for rabies virus and canine distemper virus (*11*,*12*). We assessed samples from all 3 seals for *Leptospira* infection (*13*). We subjected RNA obtained from the fox samples to sequence-independent single-primer amplification (*14*) to enable sequence generation. We then used the sequence data obtained from these samples to exclude other viral agents by removing reads that aligned with the *Vulpes vulpes* genome (*15*,*16*) and then undertaking de novo assembly to produce contiguous sequences by using SPAdes (*17*). We then screened these sequences by using custom viral databases (*18*) against the *Bornaviridae*, *Circoviridae*, *Flaviviridae*, *Herpesviridae*, *Paramyxoviridae*, *Parvoviridae*, *Pheuiviridae*, and *Rhabdoviridae* viral families.

## Results

### Clinical Setting

The swans, gray seal, and fox were housed within the quarantine unit of the center, a suite of 17 individual cubicles accessed by a central corridor (Appendix Figure 1). This facility was designed to minimize transmission of microorganisms among residents; animals in separate cubicles had no direct contact. Basic biosecurity practices were in place, such as decontamination steps between cubicles and staff using respiratory protective equipment (e.g., N95 masks) and dedicated personal protective equipment for each cubicle.

The 5 juvenile mute swans were rescued from different locations and brought to the center for treatment during October 23–November 20, 2020 (Figure 1). These birds were admitted for various reasons, including trauma and being underweight and weak. Infection with avian influenza viruses was not suspected at admission. The juvenile mute swans were housed indoors within the center’s isolation unit, mixed in groups of up to 4 with other rescued adult mute swans (Appendix Figure 1). Each of the juvenile swans had been recovering uneventfully until the sudden onset of lethargy and death or euthanasia during November 25–29 (Figure 1). Clinical signs were not observed in the remaining adult mute swans (n = 6) within the isolation unit. The isolation unit also contained 30 mallards (*Anas platyrhynchos*) housed across 3 rooms in groups of 10. No clinical disease or deaths were recorded in these birds.

The 4 common seals were estimated to be 5–6 months of age and arrived individually at the facility 1–2 months before the disease episode (Figure 1), admitted for various reasons, including poor body condition, superficial bite wounds, and lungworm. In each case, the animals had been responding well to treatment and supportive care up until the sudden onset of seizures and subsequent death or euthanasia. All 4 seals that died had been in the isolation unit (cubicles 6, 8, and 11) (Appendix Figure 1) at the time the swans (cubicles 15 and 17) were affected (Appendix Figure 1) but had subsequently been moved into another area of the facility. The affected seals had previously had close contact with other common seals in other areas of the facility; none of those animals became ill.

The gray seal was a 2-week-old pup admitted for care after maternal abandonment 2 weeks earlier (Figure 1). It was housed in the isolation unit in a cubicle opposite 1 of the swans (cubicle 1) (Appendix Figure 1). The seal was in good bodily condition and progressing well until the sudden onset of fever, facial twitching, and stupor that led to euthanasia on welfare grounds.

The fox had been in the isolation unit of the center for 2 weeks (Figure 1) in cubicle 10 (Appendix Figure 1). It had been brought to the facility with large areas of alopecia and skin crusts over the body and limbs, consistent with mange. It had been receiving treatment and was reported to be progressing well until the sudden onset of malaise and inappetence and was found dead the following morning.

### Pathologic Investigation

The body condition of the 3 swans examined ranged from good to poor. Gross findings among the carcasses included petechiae in the liver and epicardium in 1 bird and opacity of the air sacs in another. Microscopic examination of tissues from 1 bird revealed multifocal, necrotizing, nonsuppurative myocarditis, hepatitis, splenitis, nephritis, and encephalitis, along with intralesional presence of influenza A virus antigen, observed during IHC examination.

**Figure 2:**
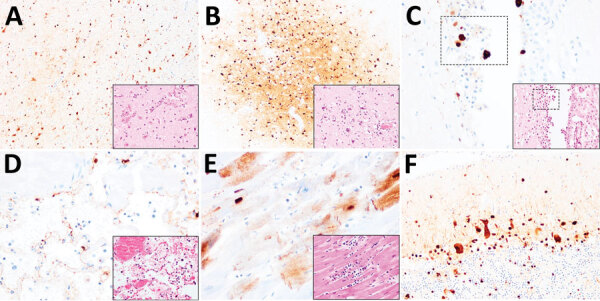
Histopathologic and immunohistochemical examination of the gray seal (*Halichoerus grypus*), common seal (*Phoca vitulina*), and red fox (*Vulpes vulpes*) infected with highly pathogenic avian influenza A subtype H5N8, United Kingdom. Serial tissue sections were stained with hematoxylin and eosin. Immunohistochemical examination was undertaken using anti–influenza A nucleoprotein primary antibody (Statens Serum Institute, https://en.ssi.dk). Insets show histopathologic study results. A) Nonsuppurative polioencephalitis and presence of virus antigens in neurons in the cerebrum, common seal (*Phoca vitulina*). Original magnification ×10, inset ×40. B) Nonsuppurative polioencephalitis with neuronophagia and association of virus antigens, red fox. Original magnification ×10, inset ×40. C) Ependymal necrosis and the association of virus antigens, fox. Original magnification and inset ×40; area of interest also shown. D) Diffuse alveolar damage and presence of virus in type I alveolar pneumocytes, red fox. Original magnification and inset ×40. E) Cardiomyonecrosis associated with virus antigens in cardiomyocytes, red fox. Original magnification and inset ×40. F) Virus antigens in granular and molecular layer of the cerebellum, red fox. Original magnification ×20, inset ×40. Serial tissue sections were stained with hematoxylin and eosin. Immunohistochemical examination was undertaken using anti–influenza A nucleoprotein primary antibody (Statens Serum Institute, https://en.ssi.dk). Insets show histopathologic study results.

The body condition of the 3 examined seals was judged to be fair. Gross examination of 2 common seals revealed generalized lymphadenomegaly and multiple pale foci in the lungs. One common seal also showed congested meninges. The gray seal pup showed generalized lymphadenomegaly but no other gross changes. Microscopic examination of the seal tissues revealed mild, eosinophilic, interstitial pneumonia in the 2 common seals, consistent with lungworm infection, and severe, necrotizing, nonsuppurative polioencephalitis in the 2 common seals and the gray seal. The lymph node sections showed nonspecific reactive hyperplasia, accounting for the gross enlargement of these organs. IHC examination for influenza A virus revealed multifocal immunolabelling in the neurons within the gray matter of the brain in all 3 seals, in close association with the inflammatory lesions (Figure 2, panel A), although virus antigen was absent in the lung, liver, kidney, and lymph node tissues of the seals.

The body condition of the fox was judged to be poor. The animal was visibly underweight, and gross examination revealed large areas of alopecia and crusts affecting the skin of the body and limbs, mild splenomegaly, and generalized reddening of the lungs. Microscopic examination of the brain, lung, and heart revealed severe, acute, nonsuppurative, polioencephalitis (Figure 2, panel B) and ventriculitis (Figure 2, panel C); severe, acute, necrotizing, nonsuppurative interstitial pneumonia (Figure 2, panel D); and mild, acute, nonsuppurative myocarditis (Figure 2, panel E). Within these lesions, IHC examination confirmed the presence of influenza virus antigens among neurons in the cerebrum (Figure 2, panel B) and cerebellum (Figure 2, panel F) and in ependymal cells (Figure 2, panel C), alveolar type I pneumocytes (Figure 2, panel D), and cardiomyocytes (Figure 2, panel E). IHC examination performed on sections of brain and lung showed no canine distemper virus.

### Virologic Assessment

Oropharyngeal and cloacal swabs from all 5 swans tested positive for H5N8 (Appendix Table). WGS data were generated for 1 of the swans submitted (A/mute swan/England/234135/H5N8 2020/12/01).

Nasal and rectal swab samples taken during PME of the 3 seals tested negative by PCR for influenza A virus. However, we retrospectively detected influenza A(H5N8) virus nucleic acid in the brain and lung of the gray seal and the brain of 2 common seals (Appendix Table). We performed virus isolation on pooled samples and successfully isolated virus from the pooled brain tissue of seals (A/seal/England/AVP-031141/2020 [H5N8]).

We detected influenza A virus RNA in each of the fox samples (brain, liver, kidney, spleen, and lung), and PCR subtyping determined the influenza A virus subtype as H5N8 (Appendix Table). We isolated virus and generated WGS from the fox brain tissue (A/red fox/England/AVP-M1-21-01/2020 (H5N8)). The hemagglutinin cleavage site (CS) motif from selected fox and seal samples had the amino acid sequence PLREKRRKRGLF, consistent with 99% of CSs characterized across highly pathogenic avian influenza (HPAI) H5N8 virus sequences from the United Kingdom during autumn and winter 2020–2021. Analysis of WGS data generated from the fox, seal, and swan samples demonstrated high sequence similarity (>99.9% nucleotide identity across all gene segments). Alignment of the viral hemagglutinin (Appendix Figure 2, panel A) and neuraminidase genes (Appendix Figure 2, panel B) demonstrated that virus from the fox and seals clustered closely with the viruses detected in the swans from the same wildlife center. Comparison of WGS data from the fox, seal, and swan identified a total of 33 aa substitutions that have been associated with altered virulence reported through natural or experimental infections. Almost all substitutions have been identified in the H5N8 virus sequences generated during the 2020–2021 epizootic in avian species (Appendix Figure 2, panel C). The genomes of the fox, seal, and swan-derived viruses were homologous at the amino-acid level, with the exception of a single amino-acid substitution at position 701 in the polymerase basic protein 2 gene (D701N) that was only present in the fox and seal sequences. Analysis of WGS data obtained from the fox samples demonstrated a lack of other viral agents, including canine distemper virus.

At the time of the outbreak, the rehabilitation center fell within a government-mandated protection zone because of previously confirmed detection of HPAI virus infections; consequently, moving birds off the center was restricted around the time of the disease episode. Restrictions were also applied specifically to the rehabilitation center when suspicion of notifiable disease in the swans was reported. The cases investigated in this outbreak were limited to animals held within the quarantine ward, and over the subsequent months, no further cases of unusual or unexplained clinical disease or death occurred among the mammals and birds at the center. After the disease event, but before diagnosis of H5N8 in the seals and fox, the center was visited as part of surveillance activities within the protection zone. Resident bird species were examined for evidence of clinical disease, and 38 birds (6 swans, 30 mallards, 1 pigeon, and 1 widgeon) were sampled for virologic testing by PCR. All birds tested negative. Blood was not taken for analysis.

## Discussion

We determined that avian-origin influenza A(H5N8) virus was the cause of death in a red fox and the cause of seizures in a gray seal and several common seals housed at a wildlife rehabilitation center. These events occurred roughly 1 week after 5 swans housed in the same quarantine unit died from infections with HPAI H5N8 virus. Genetic and epidemiologic investigations suggest that the swans were most likely the source of infection for the fox and seals; virus transmission likely occurred by fomite transfer or aerosol spread.

The severity of disease and pathologic findings in the seals and fox was unexpected. High-mortality outbreaks of disease associated with influenza A virus have been described in common seals and were associated with virus subtypes H10N7 (*19*–*21*), H7N7 (*22*), and H3N8 (*23*). In most of these cases, infection was limited to the respiratory tract. HPAI H5N8 clade 2.3.4.4b has also been detected recently in gray seals in Europe (*24*). However, the infection of a fox represents an unusual detection of this virus in association with inflammation of the central nervous system in terrestrial mammal species.

In 2016 and 2017, H5N8 virus was detected in lung tissue of 2 gray seals that were found dead, although pathologic findings typical of influenza virus infection was not reported in either carcass (*24*). The H5N8 virus of avian origin we have described was associated with acute inflammation of the central nervous system, in the absence of substantial respiratory involvement or detection in the nasal swab specimens collected before postmortem investigation; however, viral nucleic acid was indicated in the lung of the single gray seal tested. The negative result on nasal swab specimens of the seals in this outbreak is noteworthy and may have implications for surveillance for influenza in this species.

Natural infection of terrestrial carnivores with influenza A virus subtypes, although rare, has been previously reported in species such as domestic and wild felids, mustelids, and canids (*25*–*27*). Experimental infection of red foxes was previously conducted using the HPAI H5N1 subtype by intratracheal inoculation and through feeding virus-infected bird carcasses (a proxy for the putative natural route of infection) (*27*). This experimental H5N1 infection of foxes resulted in severe inflammation in the brain, lung, and heart of foxes that were challenged intratracheally, despite the animals showing no clinical signs. From the foxes fed virus-infected carcasses, only mild inflammation was observed, and it was restricted to the lungs. The study authors (Reperant et al. [*27*]) also refer in their discussion to detection of influenza A virus in foxes found dead in the field, although no further details are provided. The histopathologic findings in the intratracheally inoculated foxes are consistent with those observed in the natural infection of the fox at the rehabilitation center and would support a respiratory or airborne infection route.

In our study, evaluation of WGS data demonstrated that the virus in the swans, the fox, and the seals clustered together phylogenetically, and minimal genetic differences were observed. The WGS analysis did not enable us to determine the direction of infection; however, the epidemiologic findings combined with the genetic data generated strongly suggest that the swans were the source of the infection for the fox and seals. The D701N amino acid substitution in the polymerase basic 2 gene identified in both sequences derived from the mammalian species was absent from all avian sequences generated during this 2020–2021 outbreak in the United Kingdom. This substitution has previously been associated with mammalian adaptation and increased replicative fitness in mammalian cells (*28*–*32*). However, the substitution, in isolation, is not considered to be a factor that may result in increased avian-to-mammalian transmission risk, given that a combination of adaptive and compensatory changes observed in human sequences that would likely be required for efficient adaptation. Analysis of available H5 and H7 influenza sequences from human infections found that the D701N mutation had low prevalence and was therefore not a strong correlate for zoonotic infection. Excluding the D701N mutation, the remaining amino-acid changes identified in the fox, seal, and swan we have described were common to the H5N8 sequences obtained from the UK and Europe during the 2020–2021 epizootic. Therefore, the assessment of the sequences derived from mammalian species, when compared against both avian influenza A virus sequences from the 2020–2021 UK outbreak and sequences derived from proposed human infection, demonstrated no human risk for infection over and above that already considered for the avian isolate.

A question that remains is why infection with a highly pathogenic H5N8 isolate of avian influenza produced such severe clinical disease in mammal species in this event, although contributing factors are likely to be multifactorial and involve both virus and host. For example, a combination of underlying conditions, nutrition, and physiologic stress might have contributed to disease onset in these animals, in addition to factors associated with temporary housing of wild animals. Several of the seals were admitted to the center with lungworm infection, which is not uncommon in wild pinnipeds, and although the infections were severe enough to require treatment, several of the seals had been at the center for at least 1 month and were reported to be responding well to treatment. The affected gray seal had been admitted as a neonate 2 weeks previously and was likely to have been abandoned immediately after birth, probably without opportunity to suckle; therefore, an immature immune system and reduced passive immunity could have been contributory factors for disease. The fox was malnourished and had mange, a common infection that causes severe illness and potentially increases susceptibility to other infections, although immunosuppressive viral infections were excluded after negative results on PCR, WGS, and IHC examination. No other pathologies or disease agents were identified in the seals and fox during postmortem examination. Malnutrition is also common in young seals admitted to rehabilitation centers and was evident in the history provided for 2 of the common seals (*33*). However, a standard commercial multivitamin–multimineral supplement is given to all seals after admission and is discontinued once they are self-feeding. Furthermore, the fish used at the center is blast-frozen at sea and therefore the risk for thiaminase activity is considered low; as such, nutrition is unlikely to have played a role in predisposing the seals to infection.

The retrospective detection of influenza A virus of avian origin in these mammalian species meant that evidence of human exposure was not evaluated. However, the disease event occurred during a nationwide coronavirus disease lockdown in the United Kingdom, during which the population was required to self-monitor for signs of coronavirus disease and be tested whenever clinical disease consistent with an influenza-like illness occurred. No staff reported any illness of this type during the 6-week period after the disease event, and so we can safely conclude that staff were not infected with severe acute respiratory syndrome coronavirus 2. Use of respiratory protective equipment at the quarantine facility also would have reduced the risk for infection.

In conclusion, we determined that avian-origin influenza A(H5N8) virus caused severe disease and death in juvenile seals and a fox held in a wildlife rehabilitation center, in addition to swans that had also succumbed to the virus. All evidence suggests that the swans were the most likely source of infection for the fox and seals. The location of affected animals within the quarantine facility (Appendix Figure 1) suggests either aerosol or fomite spread as the likely cause of dissemination of infectious virus between cubicles. Although the quarantine facility is designed to limit spread of infectious microorganisms through the use of good basic hygiene practices, it is not a biosecure facility designed to handle Biosafety Level 3 pathogens; as such, highly transmissible agents such as avian influenza may well spread even with some infection prevention measures in place. Because influenza infection was not suspected at the time of the event, biosecurity practices at the center may have been less effective at preventing spread compared with those implemented at a heightened level of biosecurity, which would likely have been in place had there been an awareness of the presence of influenza infection. Determining the cause of disease in the seals and fox retrospectively was entirely reliant on collaboration between field veterinary services, pathologists, and virologists, and this case highlights the importance of wildlife disease surveillance. Although genetic analyses indicated no increased risk for human infection with the H5N8 viruses in this outbreak, the investigation shows how these viruses may have unexpected and severe health risks for mammalian species. Such spillover disease events in atypical host species constitute additional factors for veterinary authorities to consider during disease outbreaks and highlight the importance of wildlife disease surveillance that uses interdisciplinary and collaborative approaches.

Appendix Additional information about encephalitis and death in wild mammals at a rehabilitation center after systemic infection with highly pathogenic avian influenza A(H5N8), United Kingdom.
