# Supplementary material for: Encephalitis and Death in Wild Mammals at a Rehabilitation Center after Infection with Highly Pathogenic Avian Influenza A(H5N8) Virus, United Kingdom
Source: Emerg Infect Dis. 2021 Nov;27(11):2856–63. doi: 10.3201/eid2711.211225 (PMC8544989; doi:10.3201/eid2711.211225)
Supplement: Appendix — Additional information about encephalitis and death in wild mammals at a rehabilitation center after systemic infection with highly pathogenic avian influenza A(H5N8), United Kingdom. [file 21-1225-Techapp-s1.pdf]

Article DOI: https://doi.org/10.3201/eid2711.211225

Encephalitis and Death in Wild Mammals at a Rehabilitation Center after Systemic Infection with Highly Pathogenic Avian Influenza A(H5N8) Virus, United Kingdom

Appendix

Appendix Table. Results from real-time RT-PCR and virus isolation testing of clinical material collected from Mute swans, a fox and three seals

Clinical samples collected from the mute swans							
		Ct/Cq values obtained per real-time RT-PCR					
Bird no.	Swab type	M-gene	H5 HA2	N8	H5 pathotype	CS* sequencing	Interpretation
1	Cloacal	34.31†	31.92	32.36	ND‡	ND	AIV§ H5N8
2	Cloacal	35.98	34.70	35.55	ND	ND	AIV H5N8
3	Cloacal	28.67	27.90	28.09	ND	ND	AIV H5N8
4	Cloacal	36.55	ND	ND	ND	ND	AIV negative
5	Cloacal	34.05	33.62	33.45	ND	ND	AIV H5N8
1	Oropharyngeal	26.55	24.90	26.31	ND	ND	AIV H5N8
2	Oropharyngeal	31.63	30.54	31.01	ND	ND	AIV H5N8
3	Oropharyngeal	24.42	23.13	23.66	ND	ND	AIV H5N8
4	Oropharyngeal	28.83	29.72	30.06	ND	ND	AIV H5N8
5	Oropharyngeal	25.67	25.67	25.74	ND	ND	AIV H5N8
Clinical samples (tissues) collected from the seals							
Seal no. and		Ct values obtained per real-time RT-PCR					
species	Tissue source	M-gene	H5 HA2	N8	H5 pathotype	CS sequencing	Interpretation
Grey seal	Brain	29.47	27.67	25.61	26.72	ND	HPAIV H5N8
	Liver	No Ct	37.38	No Ct	No Ct	ND	AIV negative
	Lung and trachea	36.93	34.89	31.53	No Ct	ND	HPAIV H5N8
	Spleen	No Ct	38.00	No Ct	No Ct	ND	AIV negative
Common	Brain	36.25	32.86	31.53	32.38	ND	HPAIV H5N8
seal 1	Liver	No Ct	No Ct	No Ct	No Ct	ND	AIV negative
	Lung and trachea	No Ct	No Ct	No Ct	No Ct	ND	AIV negative
	Spleen	No Ct	No Ct	No Ct	No Ct	ND	AIV negative
Common	Brain	28.34	26.44	25.28	26.02	ND	HPAIV H5N8
seal 2	Liver	No Ct	No Ct	No Ct	No Ct	ND	AIV negative
	Lung and trachea	No Ct	No Ct	No Ct	No Ct	ND	AIV negative
	Spleen	No Ct	36.30	35.99	No Ct	ND	AIV H5N8
Clinical samples (tissues) collected from the red fox							
		Ct values obtained per real-time RT-PCR					
Tissue source		M-gene	H5 HA2	N8	H5 pathotype	CS sequencing	Interpretation
Brain¶		26.00	24.08	21.36	27.00	PLREKRRKRGLF	HPAIV# H5N8
Liver		32.80	29.82	27.34	31.12	ND	HPAIV H5N8
Kidney		27.70	27.50	25.46	29.86	PLREKRRKRGLF	HPAIV H5N8
Spleen		27.08	28.06	25.94	29.30	PLREKRRKRGLF	HPAIV H5N8
Lung		32.87	30.23	27.27	31.73	ND	HPAIV H5N8

*CS, cleavage site.
†Ct (threshold cycle) value less than or equal to 36.00 denote a positive result.
‡ND, not done.
§AIV, avian influenza virus.
¶Virus from the brain tissue was isolated in embryonated fowls' eggs (A/red fox/England/AVP-M1–21–01/2020 (H5N8)).
#HPAIV, highly pathogenic avian influenza virus.

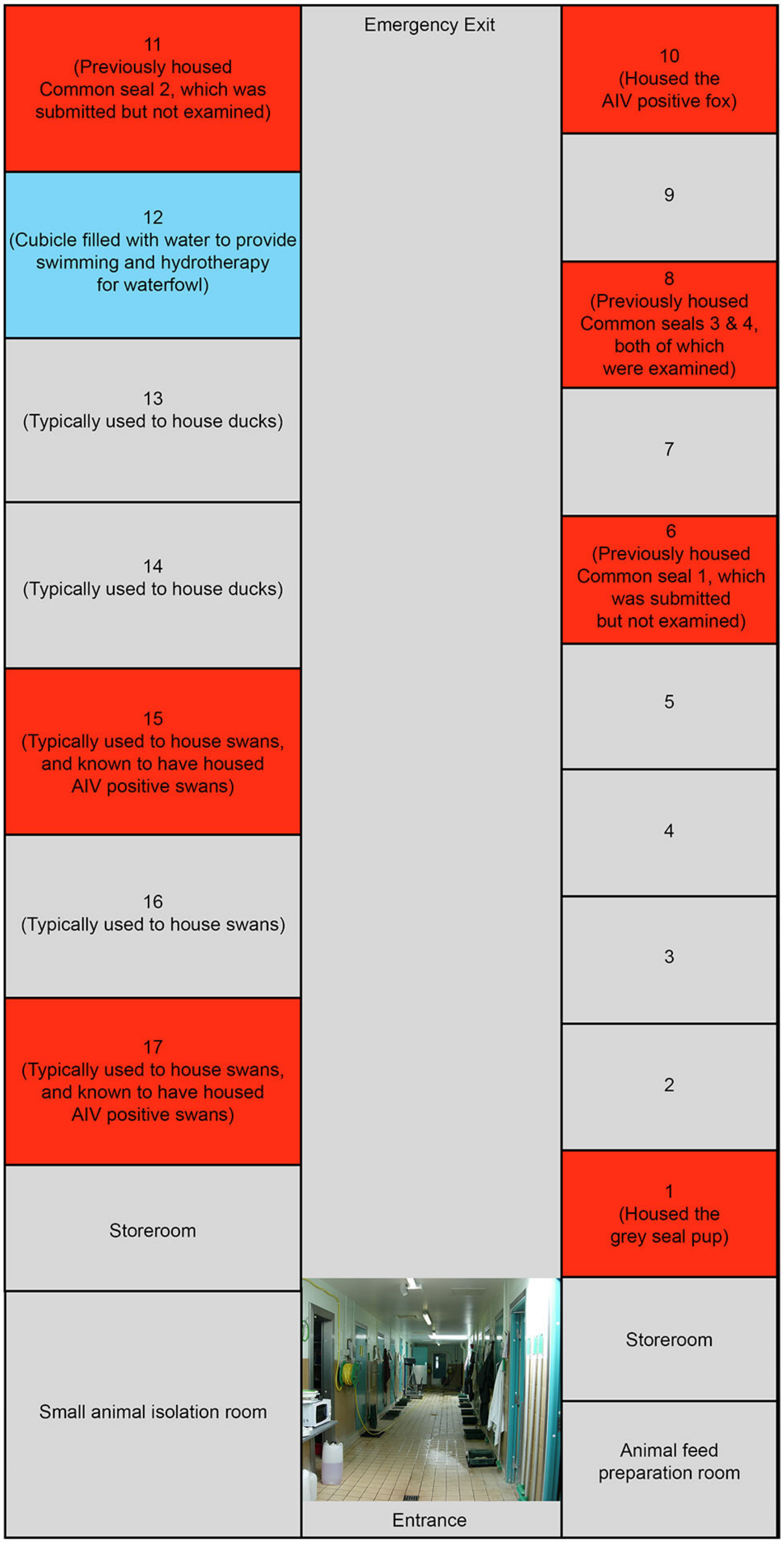

Appendix Figure 1. Schematic of the quarantine facility detailing the location of relevant species during the disease event.

A

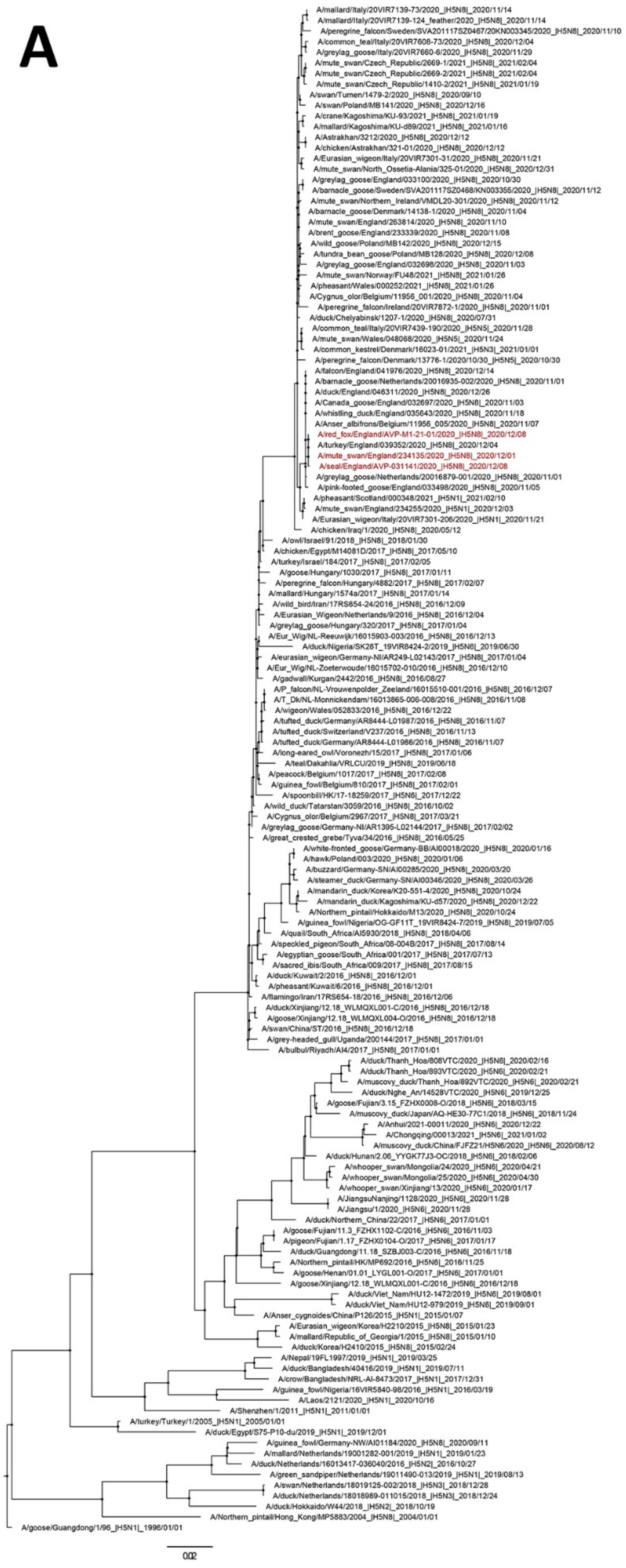
A/mallard/Italy/20VIR7139-73/2020_|H5N8|_2020/11/14
A/mallard/Italy/20VIR7139-124_feather/2020_|H5N8|_2020/11/14
A/peregrine_falcon/Sweden/SVA201117SZ0467/20KN003345/2020_|H5N8|_2020/11/10
A/common_teal/Italy/20VIR7608-73/2020_|H5N8|_2020/12/04
A/greylag_goose/Italy/20VIR7660-6/2020_|H5N8|_2020/11/29
A/mute_swan/Czech_Republic/2669-1/2021_|H5N8|_2021/02/04
A/mute_swan/Czech_Republic/2669-2/2021_|H5N8|_2021/02/04
A/mute_swan/Czech_Republic/1410-2/2021_|H5N8|_2021/01/19
A/swan/Tumen/1479-2/2020_|H5N8|_2020/09/10
A/swan/Poland/MB141/2020_|H5N8|_2020/12/16
A/crane/Kagoshima/KU-93/2021_|H5N8|_2021/01/19
A/mallard/Kagoshima/KU-d89/2021_|H5N8|_2021/01/16
A/Astrakhan/3212/2020_|H5N8|_2020/12/12
A/chicken/Astrakhan/321-01/2020_|H5N8|_2020/12/12
A/Eurasian_wigeon/Italy/20VIR7301-31/2020_|H5N8|_2020/11/21
A/mute_swan/North_Ossetia-Alania/325-01/2020_|H5N8|_2020/12/31
A/greylag_goose/England/033100/2020_|H5N8|_2020/10/30
A/barnacle_goose/Sweden/SVA201117SZ0468/KN003355/2020_|H5N8|_2020/11/12
A/mute_swan/Northern_Ireland/VMDL20-301/2020_|H5N8|_2020/11/12
A/barnacle_goose/Denmark/14138-1/2020_|H5N8|_2020/11/04
A/mute_swan/England/263814/2020_|H5N8|_2020/11/10
A/brent_goose/England/233339/2020_|H5N8|_2020/11/08
A/wild_goose/Poland/MB142/2020_|H5N8|_2020/12/15
A/tundra_bean_goose/Poland/MB128/2020_|H5N8|_2020/12/08
A/greylag_goose/England/032698/2020_|H5N8|_2020/11/03
A/mute_swan/Norway/FU48/2021_|H5N8|_2021/01/26
A/pheasant/Wales/000252/2021_|H5N8|_2021/01/26
A/Cygnus_olor/Belgium/11956_001/2020_|H5N8|_2020/11/04
A/peregrine_falcon/Ireland/20VIR7872-1/2020_|H5N8|_2020/11/01
A/duck/Chelyabinsk/1207-1/2020_|H5N8|_2020/07/31
A/common_teal/Italy/20VIR7439-190/2020_|H5N5|_2020/11/28
A/mute_swan/Wales/048068/2020_|H5N5|_2020/11/24
A/common_kestrel/Denmark/16023-01/2021_|H5N3|_2021/01/01
A/peregrine_falcon/Denmark/13776-1/2020/10/30_|H5N5|_2020/10/30
A/falcon/England/041976/2020_|H5N8|_2020/12/14
A/barnacle_goose/Netherlands/20016935-002/2020_|H5N8|_2020/11/01
A/duck/England/046311/2020_|H5N8|_2020/12/26
A/Canada_goose/England/032697/2020_|H5N8|_2020/11/03
A/whistling_duck/England/035643/2020_|H5N8|_2020/11/18
A/Anser_albifrons/Belgium/11956_005/2020_|H5N8|_2020/11/07
A/red_fox/England/AVP-M1-21-01/2020_|H5N8|_2020/12/08
A/turkey/England/039352/2020_|H5N8|_2020/12/04
A/mute_swan/England/234135/2020_|H5N8|_2020/12/01
A/seal/England/AVP-031141/2020_|H5N8|_2020/12/08
A/greylag_goose/Netherlands/20016879-001/2020_|H5N8|_2020/11/01
A/pink-footed_goose/England/033498/2020_|H5N8|_2020/11/05
A/pheasant/Scotland/000348/2021_|H5N1|_2021/02/10
A/mute_swan/England/234255/2020_|H5N1|_2020/12/03
A/Eurasian_wigeon/Italy/20VIR7301-206/2020_|H5N1|_2020/11/21
A/chicken/Iraq/1/2020_|H5N8|_2020/05/12
A/owl/Israel/91/2018_|H5N8|_2018/01/30
A/chicken/Egypt/M14081D/2017_|H5N8|_2017/05/10
A/turkey/Israel/184/2017_|H5N8|_2017/02/05
A/goose/Hungary/1030/2017_|H5N8|_2017/01/11
A/peregrine_falcon/Hungary/4882/2017_|H5N8|_2017/02/07
A/mallard/Hungary/1574a/2017_|H5N8|_2017/01/14
A/wild_bird/Iran/17RS654-24/2016_|H5N8|_2016/12/09
A/Eurasian_Wigeon/Netherlands/9/2016_|H5N8|_2016/12/04
A/greylag_goose/Hungary/320/2017_|H5N8|_2017/01/04
A/Eur_Wig/NL-Reeuwijk/16015903-003/2016_|H5N8|_2016/12/13
A/duck/Nigeria/SK28T_19VIR8424-2/2019_|H5N6|_2019/06/30
A/eurasian_wigeon/Germany-NI/AR249-L02143/2017_|H5N8|_2017/01/04
A/Eur_Wig/NL-Zoeterwoude/16015702-010/2016_|H5N8|_2016/12/10
A/gadwall/Kurgan/2442/2016_|H5N8|_2016/08/27
A/P_falcon/NL-Vrouwenpolder_Zeeland/16015510-001/2016_|H5N8|_2016/12/07
A/T_Dk/NL-Monnickendam/16013865-006-008/2016_|H5N8|_2016/11/08
A/wigeon/Wales/052833/2016_|H5N8|_2016/12/22
A/tufted_duck/Germany/AR8444-L01987/2016_|H5N8|_2016/11/07
A/tufted_duck/Switzerland/V237/2016_|H5N8|_2016/11/13
A/tufted_duck/Germany/AR8444-L01986/2016_|H5N8|_2016/11/07
A/long-eared_owl/Voronezh/15/2017_|H5N8|_2017/01/06
A/teal/Dakahlia/VRLCU/2019_|H5N8|_2019/06/18
A/peacock/Belgium/1017/2017_|H5N8|_2017/02/08
A/guinea_fowl/Belgium/810/2017_|H5N8|_2017/02/01
A/spoonbill/HK/17-18259/2017_|H5N6|_2017/12/22
A/wild_duck/Tatarstan/3059/2016_|H5N8|_2016/10/02
A/Cygnus_olor/Belgium/2967/2017_|H5N8|_2017/03/21
A/greylag_goose/Germany-NI/AR1395-L02144/2017_|H5N8|_2017/02/02
A/great_crested_grebe/Tyva/34/2016_|H5N8|_2016/05/25
A/white-fronted_goose/Germany-BB/AI00018/2020_|H5N8|_2020/01/16
A/hawk/Poland/003/2020_|H5N8|_2020/01/06
A/buzzard/Germany-SN/AI00285/2020_|H5N8|_2020/03/20
A/steamer_duck/Germany-SN/AI00346/2020_|H5N8|_2020/03/26
A/mandarin_duck/Korea/K20-551-4/2020_|H5N8|_2020/10/24
A/mandarin_duck/Kagoshima/KU-d57/2020_|H5N8|_2020/12/22
A/Northern_pintail/Hokkaido/M13/2020_|H5N8|_2020/10/24
A/guinea_fowl/Nigeria/OG-GF11T_19VIR8424-7/2019_|H5N8|_2019/07/05
A/quail/South_Africa/AI5930/2018_|H5N8|_2018/04/06
A/speckled_pigeon/South_Africa/08-004B/2017_|H5N8|_2017/08/14
A/egyptian_goose/South_Africa/001/2017_|H5N8|_2017/07/13
A/sacred_ibis/South_Africa/009/2017_|H5N8|_2017/08/15
A/duck/Kuwait/2/2016_|H5N8|_2016/12/01
A/pheasant/Kuwait/6/2016_|H5N8|_2016/12/01
A/flamingo/Iran/17RS654-18/2016_|H5N8|_2016/12/06
A/duck/Xinjiang/12.18_WLMQXL001-C/2016_|H5N8|_2016/12/18
A/goose/Xinjiang/12.18_WLMQXL004-O/2016_|H5N8|_2016/12/18
A/swan/China/ST/2016_|H5N8|_2016/12/18
A/grey-headed_gull/Uganda/200144/2017_|H5N8|_2017/01/01
A/bulbul/Riyadh/AI4/2017_|H5N8|_2017/01/01
A/duck/Thanh_Hoa/808VTC/2020_|H5N6|_2020/02/16
A/duck/Thanh_Hoa/893VTC/2020_|H5N6|_2020/02/21
A/muscovy_duck/Thanh_Hoa/892VTC/2020_|H5N6|_2020/02/21
A/duck/Nghe_An/14528VTC/2020_|H5N6|_2019/12/25
A/goose/Fujian/3.15_FZHX0008-O/2018_|H5N6|_2018/03/15
A/muscovy_duck/Japan/AQ-HE30-77C1/2018_|H5N6|_2018/11/24
A/Anhui/2021-00011/2020_|H5N6|_2020/12/22
A/Chongqing/00013/2021_|H5N6|_2021/01/02
A/muscovy_duck/China/FJFZ21/H5N6/2020_|H5N6|_2020/08/12
A/duck/Hunan/2.06_YYGK77J3-OC/2018_|H5N6|_2018/02/06
A/whooper_swan/Mongolia/24/2020_|H5N6|_2020/04/21
A/whooper_swan/Mongolia/25/2020_|H5N6|_2020/04/30
A/whooper_swan/Xinjiang/13/2020_|H5N6|_2020/01/17
A/JiangsuNanjing/1128/2020_|H5N6|_2020/11/28
A/Jiangsu/1/2020_|H5N6|_2020/11/28
A/duck/Northern_China/22/2017_|H5N6|_2017/01/01
A/goose/Fujian/11.3_FZHX1102-C/2016_|H5N6|_2016/11/03
A/pigeon/Fujian/1.17_FZHX0104-O/2017_|H5N6|_2017/01/17
A/duck/Guangdong/11.18_SZBJ003-C/2016_|H5N6|_2016/11/18
A/Northern_pintail/HK/MP692/2016_|H5N6|_2016/11/25
A/goose/Henan/01.01_LYGL001-O/2017_|H5N6|_2017/01/01
A/goose/Xinjiang/12.18_WLMQXL001-C/2016_|H5N6|_2016/12/18
A/duck/Viet_Nam/HU12-1472/2019_|H5N6|_2019/08/01
A/duck/Viet_Nam/HU12-979/2019_|H5N6|_2019/09/01
A/Anser_cygnoides/China/P126/2015_|H5N1|_2015/01/07
A/Eurasian_wigeon/Korea/H2210/2015_|H5N8|_2015/01/23
A/mallard/Republic_of_Georgia/1/2015_|H5N8|_2015/01/10
A/duck/Korea/H2410/2015_|H5N8|_2015/02/24
A/Nepal/19FL1997/2019_|H5N1|_2019/03/25
A/duck/Bangladesh/40416/2019_|H5N1|_2019/07/11
A/crow/Bangladesh/NRL-AI-8473/2017_|H5N1|_2017/12/31
A/guinea_fowl/Nigeria/16VIR5840-98/2016_|H5N1|_2016/03/19
A/Laos/2121/2020_|H5N1|_2020/10/16
A/Shenzhen/1/2011_|H5N1|_2011/01/01
A/turkey/Turkey/1/2005_|H5N1|_2005/01/01
A/duck/Egypt/S75-P10-du/2019_|H5N1|_2019/12/01
A/guinea_fowl/Germany-NW/AI01184/2020_|H5N8|_2020/09/11
A/mallard/Netherlands/19001282-001/2019_|H5N1|_2019/01/23
A/duck/Netherlands/16013417-036040/2016_|H5N2|_2016/10/27
A/green_sandpiper/Netherlands/19011490-013/2019_|H5N1|_2019/08/13
A/swan/Netherlands/18019125-002/2018_|H5N3|_2018/12/28
A/duck/Netherlands/18018989-011015/2018_|H5N3|_2018/12/24
A/duck/Hokkaido/W44/2018_|H5N2|_2018/10/19
A/Northern_pintail/Hong_Kong/MP5883/2004_|H5N8|_2004/01/01
A/goose/Guangdong/1/96_|H5N1|_1996/01/01
0.02

B

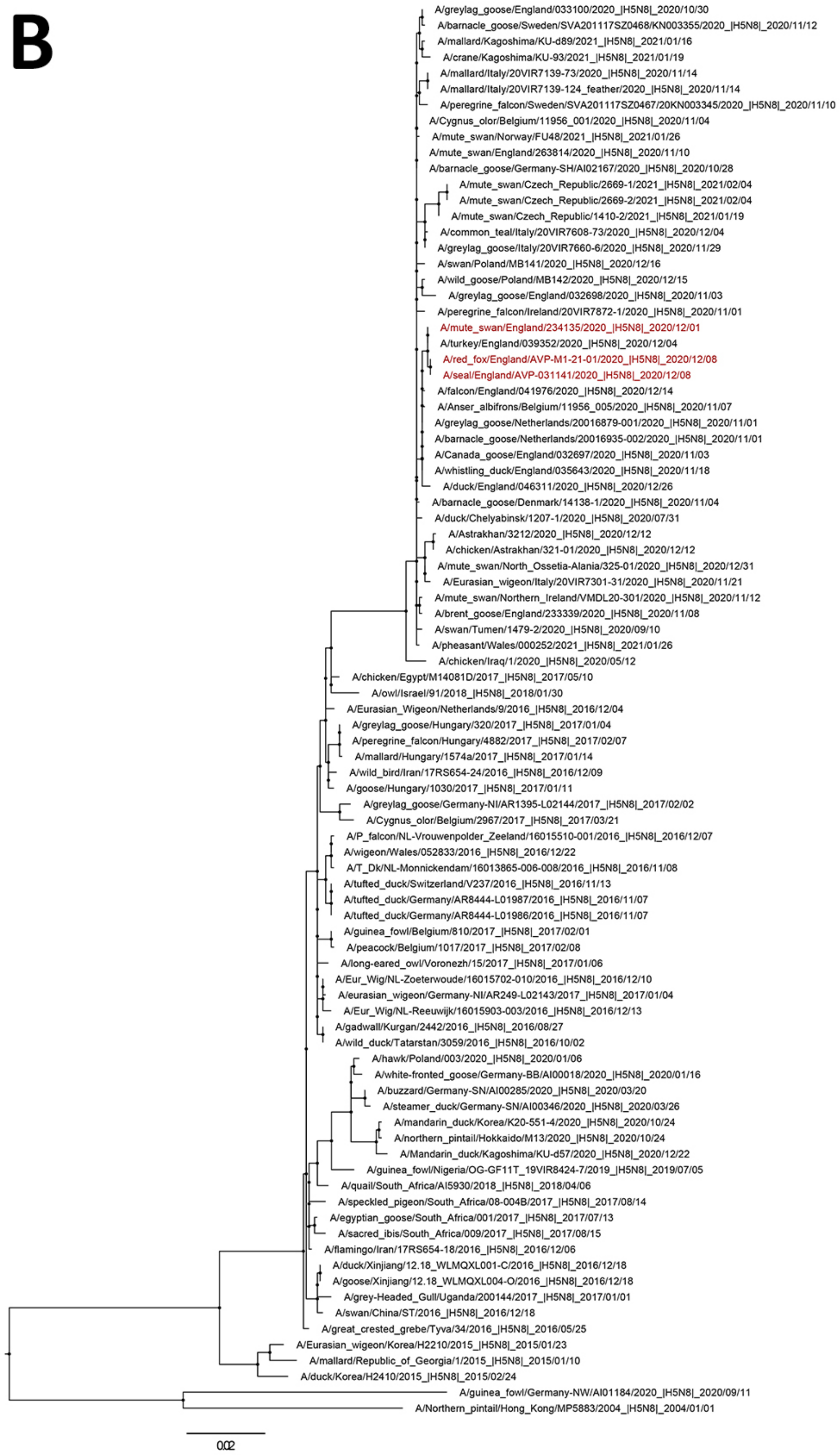

A/greylag_goose/England/033100/2020_|H5N8|_2020/10/30
A/barnacle_goose/Sweden/SVA201117SZ0468/KN003355/2020_|H5N8|_2020/11/12
A/mallard/Kagoshima/KU-d89/2021_|H5N8|_2021/01/16
A/crane/Kagoshima/KU-93/2021_|H5N8|_2021/01/19
A/mallard/Italy/20VIR7139-73/2020_|H5N8|_2020/11/14
A/mallard/Italy/20VIR7139-124_feather/2020_|H5N8|_2020/11/14
A/peregrine_falcon/Sweden/SVA201117SZ0467/20KN003345/2020_|H5N8|_2020/11/10
A/Cygnus_olor/Belgium/11956_001/2020_|H5N8|_2020/11/04
A/mute_swan/Norway/FU48/2021_|H5N8|_2021/01/26
A/mute_swan/England/263814/2020_|H5N8|_2020/11/10
A/barnacle_goose/Germany-SH/AI02167/2020_|H5N8|_2020/10/28
A/mute_swan/Czech_Republic/2669-1/2021_|H5N8|_2021/02/04
A/mute_swan/Czech_Republic/2669-2/2021_|H5N8|_2021/02/04
A/mute_swan/Czech_Republic/1410-2/2021_|H5N8|_2021/01/19
A/common_teal/Italy/20VIR7608-73/2020_|H5N8|_2020/12/04
A/greylag_goose/Italy/20VIR7660-6/2020_|H5N8|_2020/11/29
A/swan/Poland/MB141/2020_|H5N8|_2020/12/16
A/wild_goose/Poland/MB142/2020_|H5N8|_2020/12/15
A/greylag_goose/England/032698/2020_|H5N8|_2020/11/03
A/peregrine_falcon/Ireland/20VIR7872-1/2020_|H5N8|_2020/11/01
A/mute_swan/England/234135/2020_|H5N8|_2020/12/01
A/turkey/England/039352/2020_|H5N8|_2020/12/04
A/red_fox/England/AVP-M1-21-01/2020_|H5N8|_2020/12/08
A/seal/England/AVP-031141/2020_|H5N8|_2020/12/08
A/falcon/England/041976/2020_|H5N8|_2020/12/14
A/Anser_albifrons/Belgium/11956_005/2020_|H5N8|_2020/11/07
A/greylag_goose/Netherlands/20016879-001/2020_|H5N8|_2020/11/01
A/barnacle_goose/Netherlands/20016935-002/2020_|H5N8|_2020/11/01
A/Canada_goose/England/032697/2020_|H5N8|_2020/11/03
A/whistling_duck/England/035643/2020_|H5N8|_2020/11/18
A/duck/England/046311/2020_|H5N8|_2020/12/26
A/barnacle_goose/Denmark/14138-1/2020_|H5N8|_2020/11/04
A/duck/Chelyabinsk/1207-1/2020_|H5N8|_2020/07/31
A/Astrakhan/3212/2020_|H5N8|_2020/12/12
A/chicken/Astrakhan/321-01/2020_|H5N8|_2020/12/12
A/mute_swan/North_Ossetia-Alania/325-01/2020_|H5N8|_2020/12/31
A/Eurasian_wigeon/Italy/20VIR7301-31/2020_|H5N8|_2020/11/21
A/mute_swan/Northern_Ireland/VMDL20-301/2020_|H5N8|_2020/11/12
A/brent_goose/England/233339/2020_|H5N8|_2020/11/08
A/swan/Tumen/1479-2/2020_|H5N8|_2020/09/10
A/pheasant/Wales/000252/2021_|H5N8|_2021/01/26
A/chicken/Iraq/1/2020_|H5N8|_2020/05/12
A/chicken/Egypt/M14081D/2017_|H5N8|_2017/05/10
A/owl/Israel/91/2018_|H5N8|_2018/01/30
A/Eurasian_Wigeon/Netherlands/9/2016_|H5N8|_2016/12/04
A/greylag_goose/Hungary/320/2017_|H5N8|_2017/01/04
A/peregrine_falcon/Hungary/4882/2017_|H5N8|_2017/02/07
A/mallard/Hungary/1574a/2017_|H5N8|_2017/01/14
A/wild_bird/Iran/17RS654-24/2016_|H5N8|_2016/12/09
A/goose/Hungary/1030/2017_|H5N8|_2017/01/11
A/greylag_goose/Germany-NI/AR1395-L02144/2017_|H5N8|_2017/02/02
A/Cygnus_olor/Belgium/2967/2017_|H5N8|_2017/03/21
A/P_falcon/NL-Vrouwenpolder_Zeeland/16015510-001/2016_|H5N8|_2016/12/07
A/wigeon/Wales/052833/2016_|H5N8|_2016/12/22
A/T_Dk/NL-Monnickendam/16013865-006-008/2016_|H5N8|_2016/11/08
A/tufted_duck/Switzerland/V237/2016_|H5N8|_2016/11/13
A/tufted_duck/Germany/AR8444-L01987/2016_|H5N8|_2016/11/07
A/tufted_duck/Germany/AR8444-L01986/2016_|H5N8|_2016/11/07
A/guinea_fowl/Belgium/810/2017_|H5N8|_2017/02/01
A/peacock/Belgium/1017/2017_|H5N8|_2017/02/08
A/long-eared_owl/Voronezh/15/2017_|H5N8|_2017/01/06
A/Eur_Wig/NL-Zoeterwoude/16015702-010/2016_|H5N8|_2016/12/10
A/eurasian_wigeon/Germany-NI/AR249-L02143/2017_|H5N8|_2017/01/04
A/Eur_Wig/NL-Reeuwijk/16015903-003/2016_|H5N8|_2016/12/13
A/gadwall/Kurgan/2442/2016_|H5N8|_2016/08/27
A/wild_duck/Tatarstan/3059/2016_|H5N8|_2016/10/02
A/hawk/Poland/003/2020_|H5N8|_2020/01/06
A/white-fronted_goose/Germany-BB/AI00018/2020_|H5N8|_2020/01/16
A/buzzard/Germany-SN/AI00285/2020_|H5N8|_2020/03/20
A/steamer_duck/Germany-SN/AI00346/2020_|H5N8|_2020/03/26
A/mandarin_duck/Korea/K20-551-4/2020_|H5N8|_2020/10/24
A/northern_pintail/Hokkaido/M13/2020_|H5N8|_2020/10/24
A/Mandarin_duck/Kagoshima/KU-d57/2020_|H5N8|_2020/12/22
A/guinea_fowl/Nigeria/OG-GF11T_19VIR8424-7/2019_|H5N8|_2019/07/05
A/quail/South_Africa/AI5930/2018_|H5N8|_2018/04/06
A/speckled_pigeon/South_Africa/08-004B/2017_|H5N8|_2017/08/14
A/egyptian_goose/South_Africa/001/2017_|H5N8|_2017/07/13
A/sacred_ibis/South_Africa/009/2017_|H5N8|_2017/08/15
A/flamingo/Iran/17RS654-18/2016_|H5N8|_2016/12/06
A/duck/Xinjiang/12.18_WLMQXL001-C/2016_|H5N8|_2016/12/18
A/goose/Xinjiang/12.18_WLMQXL004-O/2016_|H5N8|_2016/12/18
A/grey-Headed_Gull/Uganda/200144/2017_|H5N8|_2017/01/01
A/swan/China/ST/2016_|H5N8|_2016/12/18
A/great_crested_grebe/Tyva/34/2016_|H5N8|_2016/05/25
A/Eurasian_wigeon/Korea/H2210/2015_|H5N8|_2015/01/23
A/mallard/Republic_of_Georgia/1/2015_|H5N8|_2015/01/10
A/duck/Korea/H2410/2015_|H5N8|_2015/02/24
A/guinea_fowl/Germany-NW/AI01184/2020_|H5N8|_2020/09/11
A/Northern_pintail/Hong_Kong/MP5883/2004_|H5N8|_2004/01/01
0.02

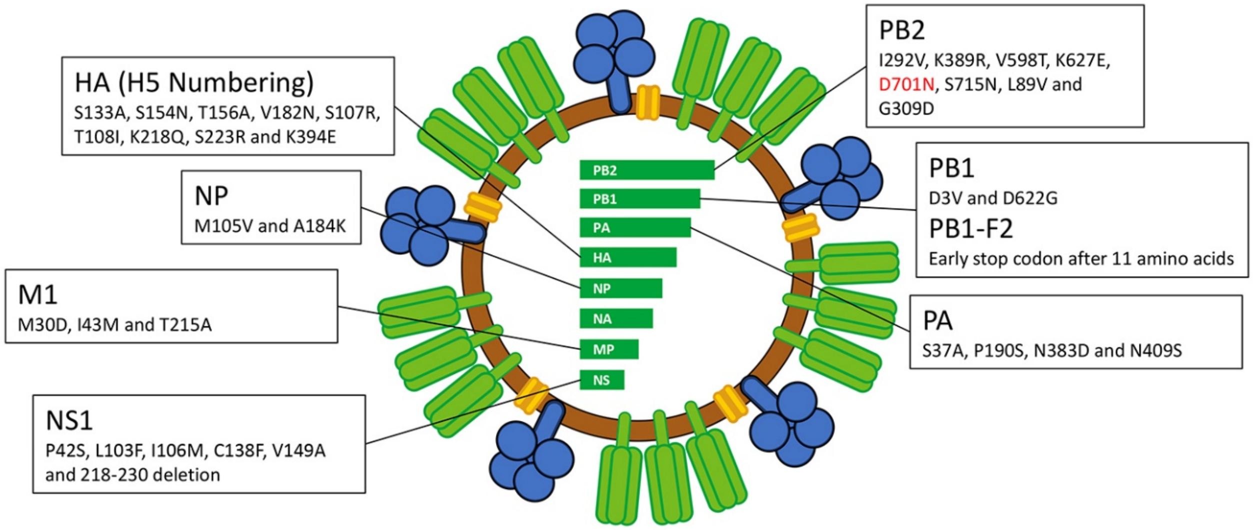

Appendix Figure 2. Genetic analyses of HA and NA from samples characterized from the disease event. A) Maximum-likelihood (ML) phylogenetic tree inferred from HA H5Nx segments predominantly from wild bird hosts. B) ML phylogenetic tree inferred from the NA segment of H5N8 viruses. All sequences generated in this study are colored red; C) Viral schematic detailing amino acid changes present in the fox, seal and swan-derived viruses. Substitutions previously associated with altered phenotype in the literature are listed by gene segment. The D701N mutation detected only in the mammalian sequences is highlighted in red. Only H5 sequences from wild birds or poultry that were ancestral to the latest epizootic or contemporaneously detected in UK poultry outbreaks were included. Sequences were aligned with MAFFT v7.450 and trimmed to starting ATG and ending STOP codon using Aliview v1.26. Maximum-likelihood phylogenetic trees were generated with IQ-TREE v1.6.12 and branch supports were obtained with Shimodaira-Hasegawa-like approximate Likelihood-Ratio-Test (aLRT) with 1000 iterations. The final trees were visualized and annotated using FigTree v1.4.3 (https://github.com/rambaut/figtree) rooted through the outgroup A/Northern_pintail/Hong_Kong/MP5883/2004 and nodes placed in ascending order. The PDF is zoomable.
